# Quantifying the impact of pre-existing conditions on the stage of oesophagogastric cancer at diagnosis: a primary care cohort study using electronic medical records

**DOI:** 10.1093/fampra/cmaa132

**Published:** 2020-12-21

**Authors:** Myra Quiroga, Elizabeth A Shephard, Luke T A Mounce, Madeline Carney, William T Hamilton, Sarah J Price

**Affiliations:** 1 Morsani College of Medicine, University of Southern Florida, Tampa, FL, USA; 2 Discovery Research Group, College of Medicine and Health, University of Exeter, St Luke’s Campus, Exeter, UK

**Keywords:** Cancer care/oncology, cancer epidemiology, doctor–patient relationship, electronic medical records, medical comorbidity, primary care

## Abstract

**Background:**

Pre-existing conditions interfere with cancer diagnosis by offering diagnostic alternatives, competing for clinical attention or through patient surveillance.

**Objective:**

To investigate associations between oesophagogastric cancer stage and pre-existing conditions.

**Methods:**

Retrospective cohort study using Clinical Practice Research Datalink (CPRD) data, with English cancer registry linkage. Participants aged ≥40 years had consulted primary care in the year before their incident diagnosis of oesophagogastric cancer in 01/01/2010–31/12/2015. CPRD records pre-diagnosis were searched for codes denoting clinical features of oesophagogastric cancer and for pre-existing conditions, including those providing plausible diagnostic alternatives for those features. Logistic regression analysed associations between stage and multimorbidity (≥2 conditions; reference category: no multimorbidity) and having ‘diagnostic alternative(s)’, controlling for age, sex, deprivation and cancer site.

**Results:**

Of 2444 participants provided, 695 (28%) were excluded for missing stage, leaving 1749 for analysis (1265/1749, 72.3% had advanced-stage disease). Multimorbidity was associated with stage [odds ratio 0.63, 95% confidence interval (CI) 0.47–0.85, *P* = 0.002], with moderate evidence of an interaction term with sex (1.76, 1.08–2.86, *P* = 0.024). There was no association between alternative explanations and stage (odds ratio 1.18, 95% CI 0.87–1.60, *P* = 0.278).

**Conclusions:**

In men, multimorbidity is associated with a reduced chance of advanced-stage oesophagogastric cancer, to levels seen collectively for women.

## Lay Summary

Diagnosing cancer is complicated by existing medical conditions. Diagnosis may be delayed if conditions explain cancer symptoms, or dominate appointments. Diagnosis may be quicker if conditions increase doctor–patient contact.

We studied the association between existing illness and stage (early or advanced) of diagnosis with cancer of the stomach or gullet. We studied the primary-care records of patients aged ≥40 years, diagnosed in 01/01/2010–31/12/2015, and got stage from English cancer registry data. We searched the primary-care records for cancer symptoms (e.g. difficulty swallowing), and for 27 conditions that were common or explained cancer symptoms (e.g. difficulty swallowing following a stroke). We analysed cancer stage, looking at age, sex, multimorbidity (two or more conditions) and explanations for symptoms. We studied 1749 patients, of whom 1265 (72.3%) had advanced-stage cancer. The chance of advanced stage was similar in women with (71%, 95% CI 66–75%) or without (69%, 62–76%) multimorbidity. It was lower for men with (70%, 67–74%) than without (79%, 75–83%) multimorbidity. Stage of cancer was not affected by having explanations for cancer symptoms. In summary, for men, multimorbidity is associated with a reduced chance of advanced-stage cancer of the stomach or gullet to levels seen collectively for women.

**Key Messages**

- First study of association between multimorbidity and oesophagogastric cancer stage.
- In men, multimorbidity is associated with reduced chance of advanced stage.
- This study could not investigate the mechanism of this association.
- Having diagnostic alternatives for cancer symptoms was not associated with stage.

## Introduction

Diagnosing cancer early is a UK government priority (1). By 2028, the target is for 75% of UK cancers to be diagnosed at an early stage, leading to 55 000 more people annually surviving 5 years post-diagnosis (1). Recognizing cancer symptoms and prompt investigation are key to achieving this.

In 2015–17, there were 15 800 annual diagnoses of oesophageal (*n* = 9200) or stomach (*n* = 6600) cancers, with 12 300 deaths (oesophagus, *n* = 7900; stomach, *n* = 4400) (2,3). Oesophageal and gastric cancers are generally considered together in diagnostic studies because they share symptoms and investigation pathways (4). Approximately two-thirds of diagnoses are in men (2,3). Women have a higher risk of emergency presentation and poorer 5- and 10-year survival than men (5), although their 1-year survival is similar (2,3).

Around 50–60% of all cases are advanced-stage diagnoses (note incomplete staging data levels of 19% for oesophageal, 27% for stomach cancers), requiring more intense treatment, and with poorer outcomes (2,3). Diagnostic delay may contribute (6). Compared with rectal cancer, the odds of requiring three or more pre-referral primary-care consultations was higher for gastric [1.96, 95% confidence interval (CI) 1.65–2.34] and oesophageal (1.15, 0.98–1.36) cancers (7). This suggests the potential for earlier investigation in the disease process. A shorter diagnostic interval (time from first reported symptom to diagnosis) is associated with early-stage diagnosis of oesophageal cancer (8).

Patients with two or more pre-existing medical conditions (i.e. multimorbidity) may require complex management, with separate clinical pathways for each condition (9,10). This could expedite or delay diagnosis. Expedited diagnosis may occur in patients whose conditions require routine monitoring, by increasing opportunities for symptom reporting (the surveillance hypothesis) (11–13). In contrast, diagnosis may be delayed if cancer symptoms mimic those of the pre-existing condition (the alternative-explanations hypothesis) (13,14), or the primary-care consultations are dominated by condition management (the competing-demands hypothesis) (13).

Increased pre-existing condition count is associated with advanced-stage cancers of the ovary (15), larynx (16) and breast (12). In colorectal cancer, diagnostic intervals were longer by 32 days in patients with four or more conditions (17). Patients and clinicians may be more likely to normalize pre-existing symptoms, such as rectal bleeding, especially in patients with gastrointestinal conditions (18). One study investigating pre-existing multimorbidity in nine cancers found an increased chance of advanced-stage diagnosis in those patients with three or more conditions (19). Patients with upper gastrointestinal cancer (liver/gastric) had the highest cancer-specific index of multimorbidity (26%) of all nine cancers (19).

The impact of pre-existing conditions on the stage of oesophagogastric cancer at diagnosis is unknown. This study aimed to investigate whether oesophagogastric cancer stage is associated with: (i) pre-existing conditions that share symptoms of oesophagogastric cancer (‘alternative-explanations’ hypothesis) and (ii) multimorbidity (‘competing-demands’ or ‘surveillance’ hypotheses).

## Methods

This retrospective cohort study of participants diagnosed with oesophagogastric cancer between 1 January 2010 and 31 December 2015 was set in primary care in England. Data sources were the UK’s Clinical Practice Research Datalink (CPRD) GOLD database, with linkage to Public Health England’s National Cancer Registration and Analysis Service (NCRAS, set 15) and Office for National Statistics (ONS) data. The CPRD holds anonymized longitudinal electronic records on symptoms, diagnoses, prescriptions and investigations of over 11.3 million patients from 674 UK general practices (20). Linked NCRAS data verified the date and type of diagnosis, and provided stage. Linked deprivation data (Townsend score) were obtained from ONS.

### Inclusion and exclusion criteria

Participants were selected if they:

1. Were aged ≥40 years.
2. Had an incident oesophagogastric cancer code (International Classification of Diseases, version 10 codes C15, C16) in NCRAS between 1 January 2010 and 31 December 2015.
3. Had attended their GP at least once in the year before diagnosis.

Participants were excluded for missing NCRAS data on the best estimate of stage based on tumour size, nodal involvement and the presence of metastases (21).

### Patient characteristics

Patient age and sex were provided by CPRD variables, assigning a birthday of 1 July. Patients were categorized from 1 (least deprived) to 5 (most deprived) using Townsend deprivation score data.

### Stage and date at diagnosis

Stage at diagnosis was the outcome variable for regression analysis. Stage was classified as early (stage 1 or 2) or advanced (stage 3 or 4) using the NCRAS staging variable. Diagnosis date was provided by NCRAS.

### Features of possible oesophagogastric cancer before diagnosis

Participants’ CPRD records in the year before diagnosis were searched for codes for features of possible oesophagogastric cancer: dysphagia, dyspepsia, reflux, weight loss, upper abdominal pain, nausea, vomiting, haematemesis and anaemia (4). Relevant Read code lists were assembled using an established protocol (22). Participant-level variables described the first possible feature of cancer (hereafter called ‘index feature’), with category ‘0’ denoting asymptomatic status. The ‘index date’ was the date of the index feature, or the diagnosis date for asymptomatic participants.

### Pre-existing conditions

Participant CPRD records before the index date were examined for medical codes for: (i) chronic conditions in the UK Quality and Outcomes Framework (QOF) (23); and (ii) conditions sharing symptoms with oesophagogastric cancer (selected by MQ, MC and WH) (Table 1).

**Table 1.** Conditions providing alternative diagnostic explanations for features of possible oesophageal cancer

Feature of possible oesophageal cancer	Condition providing an alternative diagnostic explanation for the feature
Dysphagia	Parkinson’s disease, oesophageal stricture and stroke (also a QOF condition)
Weight loss and anaemia	Inflammatory bowel disease and chronic kidney disease (also a QOF condition)
Nausea, vomiting and upper abdominal pain	Hernia, pancreatitis, ulcer, gastritis, oesophagitis and irritable bowel syndrome
Haematemesis	Anticoagulant medications (note, the need for anticoagulation was treated as a ‘condition’)
Dyspepsia/reflux	Oesophagitis, gastritis

The QOF conditions were: anxiety and/or depression; asthma; atrial fibrillation; chronic obstructive pulmonary disease; coronary heart disease; dementia; diabetes mellitus; epilepsy; heart failure; high blood pressure; hypothyroidism; learning disability; mental health disorders (bipolar disorder, schizophrenia and other psychoses); osteoporosis; peripheral arterial disease and rheumatoid arthritis.

### Explanatory variables denoting multimorbidity and alternative explanations

Participant-level indicator variables identified participants with two or more pre-existing conditions (i.e. multimorbidity) before the index date. The numbers (percentages) of participants (by sex) with each pre-existing condition are reported.

Participant-level indicator variables denoting ‘alternative explanations’ identified participants with one or more pre-existing conditions that provided a plausible explanation for their index feature(s).

### Data analysis

The cohort is summarized using descriptive statistics. The numbers with each comorbid condition and with alternative explanations are reported by sex, for all participants included in analysis or excluded for missing stage. Associations with sex were explored using the chi-square test.

Associations between stage at diagnosis (reference group: early stage) and both multimorbidity (reference group: no multimorbidity) and the presence of alternative explanations (reference group: no alternative explanations) were analysed using multi-level logistic regression. Interaction terms were sought on clinical grounds between sex and: (i) multimorbidity, and (ii) the presence of an alternative explanation; and (iii) between multimorbidity and age. The analyses accounted for the correlation among individuals within the same general practices (clusters) and adjusted for possible confounding by age at diagnosis, sex, deprivation and cancer site. Robust standard errors accounted for heteroscedasticity and the analysis was repeated with log(age) and with age-squared to check for non-linearity. Post-estimation diagnostics tested for model specification (linktest), goodness of fit (lfit) and collinearity between explanatory variables (collin, Collinearity Diagnostics, Philip B Ender, UCLA Office of Academic Computing).

The regression coefficients are presented. To ease interpretation, after running the final model, we used Stata’s margins commands to estimate the predicted probability of having an advanced-stage diagnosis for the following groups of interest, holding other variables to their mean values:

1. Men and women with multimorbidity.
2. Men and women with alternative explanations.

Data analysis was conducted using Stata (version 16) (StataCorp, College Station, TX).

### Missing data, bias and sensitivity analysis

Both CPRD and NCRAS have missing data. In line with convention, we interpreted the absence of a code for a clinical event as its non-occurrence (14,20,24). We classified participants with no recorded features of cancer and/or no diagnostic codes for comorbid conditions as not presenting with alternative explanations.

To examine for bias, we conducted a logistic regression to compare participants with and without staging data with regard to the explanatory variables for our main model, including interaction terms between multimorbidity and age or sex.

In sensitivity analysis, we re-ran the final model, including participants with missing staging data assigned to advanced-stage diagnosis.

### Power calculation

The complete-case sample of 1749 patients had >95% power to detect a change of 10 percentage points in the proportion diagnosed with advanced disease between those with and without multimorbidity (two-sided, alpha = 0.01). This is based on 60% of participants being multimorbid and 70% having advanced-stage disease (9,10).

## Results

**Figure 1.**
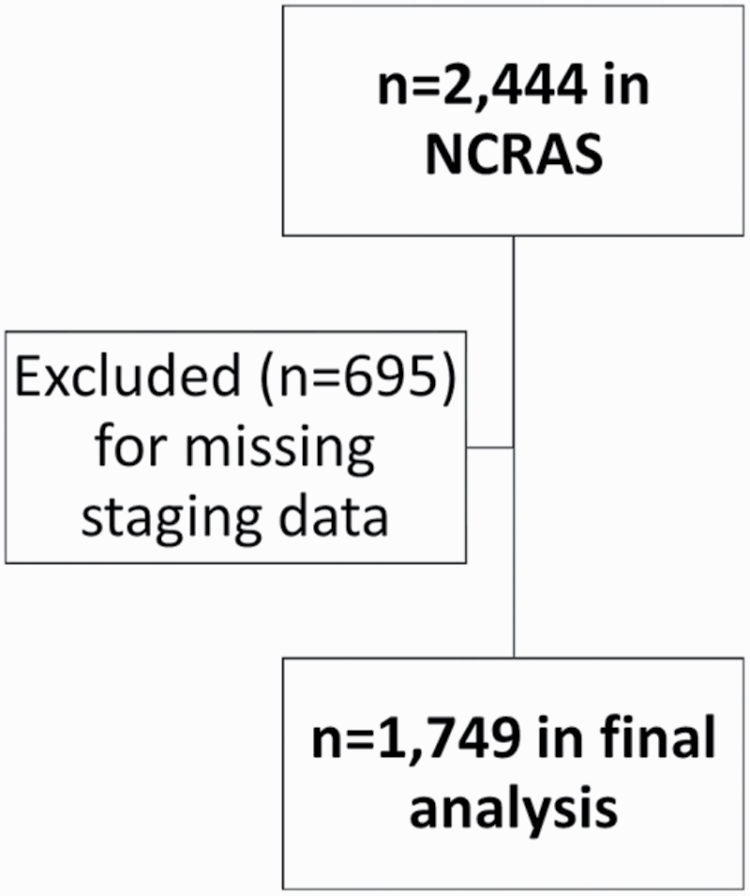
Exclusion criteria.

The NCRAS-linked CPRD GOLD dataset provided 2444 participants meeting the inclusion criteria (Table 2). Six hundred and ninety-five (28.4%) participants (*n* = 268, 38.6% female) were excluded for missing stage (Fig. 1), leaving 1749 (513 female, 29.3%) participants (oesophagus: *n* = 1089, 62.3%; stomach: *n* = 660, 37.7%) in the analysis. The mean age at diagnosis was 69.2 (SD 10.4) years for men and 72.4 (12.2) years for women. The mean (SD) age of excluded participants was 75.4 (12.0) years [male: 73.3 (11.3) years, *n* = 427; female: 78.9 (12.3) years, *n* = 268]. The median time from index date to diagnosis date was 24 days (interquartile range 6–89 days).

**Table 2.** Characteristics of study participants (included or excluded for missing stage)

Covariate	Included in analysis (*N* = 1749)			Excluded for missing stage (*N* = 695)
	Early stage	Advanced stage	Total in analysis	
*N* (%)	484 (27.7)	1265 (72.3)	1749	695
Female, *n* (%)	157 (32.4)	356 (28.1)	513 (29.3)	268 (38.6)
Age, mean (SD)	71.8 (10.9)	69.5 (11.1)	70.1 (11.1)	75.4 (12.0)
Site				
Oesophagus, *n* (%)	285 (58.9)	804 (63.6)	1089 (62.3)	394 (56.7)
Stomach, *n* (%)	199 (41.1)	461 (36.4)	660 (37.7)	301 (43.3)
Townsend quintile, mean (SD)	2.8 (1.3)	2.9 (1.3)	2.9 (1.3)	2.8 (1.3)
Presented with feature of possible cancer, *n* (%)	374 (77.3)	1013 (80.1)	1387 (79.3)	532 (76.6)
Multimorbidity, *n* (%)	344 (71.1)	792 (62.6)	1136 (65.0)	490 (70.5)
Alternative explanation, *n* (%)	81 (16.7)	199 (15.7)	280 (16.0)	123 (17.7)

Participants were aged ≥40 years and had attended their CPRD general practice at least once in the year before their incident diagnosis with oesophagogastric cancer in the period 1 January 2010 to 31 December 2015. Participants with features of possible cancer presented with any of dysphagia, dyspepsia and/or reflux, weight loss, upper abdominal pain, nausea, vomiting, haematemesis or anaemia in the year before diagnosis. Multimorbidity was defined as having at least two conditions diagnosed before either the first possible feature of cancer or cancer diagnosis (for participants who did not present with cancer features). Participants with alternative explanations had pre-existing conditions providing a plausible diagnostic alternative for their cancer feature.

### Index features of cancer

Most included participants (1387/1749, 79.3%) had recorded features of possible oesophagogastric cancer before diagnosis (Table 2). Dysphagia was most common in men (324/1236, 26.2%) and women (159/513, 31.0%). Dyspepsia and/or reflux was the next most common (men: 231/1236, 18.7%; women: 83/513, 16.8%), followed by anaemia (194/1236, 15.7%; 69/513, 13.5%) and upper abdominal pain (143/1236, 11.6%; 63/513, 12.3%). Weight loss (50/1236, 4.0%; 18/513, 3.5%), vomiting (25/1236, 2.0%; 44/513, 8.6%), nausea (42/1236, 3.4%; 12/513, 2.3%) and haematemesis (11/1236, 0.9%; 1/513, 0.2%) were relatively infrequent.

A similar proportion of excluded participants (532/695, 76.6%) had pre-diagnostic features of oesophagogastric cancer (see Supplementary Material).

### Comorbid conditions

Most participants in the analysis (1136/1749, 65.0%) were classified as multimorbid before the index date: 344/484 (71.1%) in those with early-stage and 792/1265 (62.6%) in advanced-stage disease (Table 2). The most common conditions were hypertension (43.5% in men, 45.6% in women) and being on anticoagulant medication (43.3% men, 39.0% women) (see Table 3). Most conditions had an even sex distribution, with some exceptions of conditions predominant in women; notably, anxiety/depression (21.6% in men, 36.8% in women), hypothyroidism (4.1% in men, 15.8% in women), osteoporosis (1.1% in men, 12.1% in women) and irritable bowel syndrome (4.0% in men, 9.9% in women).

**Table 3.** Numbers (%) of males and females with each comorbidity

Comorbidity	With staging data			Missing staging data		
	Males, *n* (% of 1236 males in analysis)	Females, *n* (% of 513 females in analysis)	*P* value	Males, *n* (% of 427 excluded from analysis)	Females, *n* (% of 268 excluded from analysis)	*P* value
Anticoagulants	535 (43.3)	200 (39.0)	0.097	216 (50.6)	133 (49.6)	0.806
**Anxiety/depression**	267 (21.6)	189 (36.8)	<0.0001	83 (19.4)	89 (33.2)	<0.0001
Asthma	126 (10.2)	64 (12.5)	0.163	39 (9.1)	32 (11.9)	0.234
Atrial fibrillation	82 (6.6)	35 (6.8)	0.886	43 (10.1)	31 (11.6)	0.533
**CHD**	227 (18.4)	66 (12.9)	0.005	79 (18.5)	32 (11.9)	0.022
**CKD**	174 (14.1)	94 (18.3)	0.025	68 (15.9)	63 (23.5)	0.013
COPD	113 (9.1)	38 (7.4)	0.240	47 (11.0)	25 (9.3)	0.480
**Diabetes**	219 (17.7)	54 (10.5)	<0.0001	84 (19.7)	37 (13.8)	0.047
Gastritis/oesophagitis	225 (18.2)	95 (18.5)	0.877	66 (15.5)	48 (17.9)	0.395
Heart failure	44 (3.6)	21 (4.1)	0.591	22 (5.2)	17 (6.3)	0.507
**Hernia**	98 (7.9)	62 (12.1)	0.006	31 (7.3)	36 (13.4)	0.007
Hypertension	538 (43.5)	234 (45.6)	0.424	191 (44.7)	148 (55.2)	0.007
**Hypothyroidism**	50 (4.1)	81 (15.8)	<0.0001	19 (4.5)	40 (14.9)	<0.0001
IBD	68 (5.5)	34 (6.6)	0.360	17 (4.0)	14 (5.2)	0.440
**IBS**	49 (4.0)	51 (9.9)	<0.0001	12 (2.8)	21 (7.8)	0.002
**Osteoporosis**	13 (1.1)	62 (12.1)	<0.0001	11 (2.6)	39 (14.6)	<0.0001
Peripheral arterial disease	62 (5.0)	18 (3.5)	0.170	35 (8.2)	17 (6.3)	0.366
**Rheumatoid arthritis**	21 (1.7)	18 (3.5)	0.020	5 (1.2)	6 (2.2)	0.272
Schizophrenia, bipolar disorder, other psychoses	16 (1.3)	2 (0.4)	0.088	4 (0.9)	8 (3.0)	0.044
Stroke	113 (9.1)	44 (8.6)	0.706	42 (9.8)	26 (9.7)	0.954
**Ulcer**	76 (6.2)	16 (3.1)	0.010	33 (7.7)	14 (5.2)	0.201
Other	48 (3.9)	22 (4.3)	0.694	30 (7.0)	31 (11.6)	0.039

CHD, coronary heart disease; CKD, chronic kidney disease; COPD, chronic obstructive pulmonary disease; IBD, inflammatory bowel disease; IBS, irritable bowel syndrome. Other conditions in men and women, respectively: epilepsy (*n* = 27, *n* = 7); dementia (*n* = 10, *n* = 10); Parkinson’s (*n* = 4, *n* = 1); pancreatitis (*n* = 1, *n* = 1); oesophageal stricture (*n* = 2 men); learning disability (*n* = 4, *n* = 3).

*P* values are from chi-square tests. Gender differences for participants included in analysis highlighted in bold.

The majority of participants excluded for missing stage were classified as multimorbid (490/695, 70.5%) (Table 3).

### Alternative explanations

Alternative explanations for index features of cancer occurred in 280/1749 (16.0%) included participants (123/695, 17.7% excluded participants), with similar patterns in men and women. The most common combinations were alternative explanations for nausea, vomiting or upper abdominal pain (included participants: 92/1749, 5.3%; excluded participants: 45/695, 6.5%), for weight loss or anaemia (88/1749, 5.0%; 46/695, 6.6%) and for dyspepsia/reflux (56/1749, 3.2%; 14/695, 2.0%).

### Stage at diagnosis

Advanced-stage oesophagogastric cancer was diagnosed in 1265/1749 (72.3%) of included participants (909/1236, 73.5% for men; 356/513, 69.4% for women). The proportion with multimorbidity was higher in those with early-stage (71.1%) than with advanced-stage (62.6%) disease. The proportions with alternative explanations for their index features of cancer were similar in patients with early or with advanced-stage disease (Table 2).

### Regression analyses

In univariable analyses, multimorbidity (odds ratio 0.68, 95% CI 0.55–0.85, *P* = 0.001) and age at diagnosis (0.98, 0.97–0.99, *P* < 0.0001) were associated with stage at diagnosis. In multivariable analyses, there was moderate evidence of an interaction term between sex and multimorbidity (odds ratio 1.76, 95% CI 1.08–2.86, *P* = 0.024, Table 4), controlling for age, deprivation and cancer site.

**Table 4.** Logistic regression analyses of stage at diagnosis (early versus advanced), reporting odds ratios (95% CIs) for unadjusted and adjusted analyses

Covariate	Unadjusted (univariable analysis)		Adjusted (multivariable analysis)	
	OR (95% CI)	*P* value	OR (95% CI)	*P* value
Multimorbidity	0.68 (0.55–0.85)	0.001	0.63 (0.47–0.85)	0.002
Sex	0.82 (0.65–1.03)	0.083	0.59 (0.39–0.89)	0.011
Interaction term (multimorbidity × sex)	n/a	n/a	1.76 (1.08–2.86)	0.024
Has an ‘alternative explanation’	0.93 (0.70–1.22)	0.600	1.07 (0.80–1.43)	0.631
Cancer site	1.22 (1.00–1.50)	0.068	1.21 (0.97–1.50)	0.090
Age at diagnosis	0.98 (0.97–0.99)	<0.0001	0.99 (0.98–0.99)	0.004
Deprivation quintile	1.03 (0.96–1.11)	0.399	1.04 (0.96–1.13)	0.304

Multimorbidity was defined as ≥2 pre-existing conditions. Participants with ‘alternative explanations’ had a pre-existing condition that provided a plausible diagnostic alternative for their presenting feature of oesophagogastric cancer (i.e. dysphagia, dyspepsia and/or reflux, weight loss, upper abdominal pain, nausea, vomiting, haematemesis or anaemia). *Note*: Odds ratios (ORs) >1 indicate increased odds of advanced stage at diagnosis and ORs <1 indicate reduced odds. An ‘alternative explanation’ is a condition that provides a plausible diagnostic alternative for the index cancer symptom (reference group: no alternative explanation). Multimorbidity is defined as two or more pre-existing conditions (reference group: not multimorbid). For sex, the reference group is men.

No interaction terms were found between age and multimorbidity (1.00, 0.98–1.02, *P* = 0.682) or between sex and alternative explanations (1.69, 0.64–2.12, *P* = 0.608).

Post-estimation margins commands on the final model reported the probability of advanced-stage diagnosis to be similar in women with (0.71, 0.66–0.75, *P* < 0.0001, *n* = 353) or without (0.69, 0.62–0.76, *P* < 0.0001, *n* = 160) multimorbidity. The probability of advanced-stage diagnosis in men was lower for those with multimorbidity (0.70, 0.67–0.74, *P* < 0.0001, *n* = 783) than in those without (0.79, 0.75–0.83, *P* < 0.0001, *n* = 453).

Having alternative explanations was not associated with stage (Table 4). Consequently, the probabilities of advanced-stage disease were similar in people with (0.74, 0.69–0.78) and without (0.72, 0.70–0.75) alternative explanations.

Post-estimation regression diagnostics suggested no problems in model specification, goodness of fit or collinearity between explanatory variables (results available from authors).

### Missing data and sensitivity analyses

In logistic regression comparing patients with and without staging data, missingness was associated with increasing age (1.04, 95% CI 1.03–1.05, *P* < 0.0001) and with being female (1.28, 1.06–1.55, *P* = 0.01). We found no interactions between multimorbidity and age (interaction term: 1.02, 95% CI 0.99–1.04, *P* = 0.12) or sex (1.28, 0.85–1.90, *P* = 0.251).

In ‘missing-is-advanced-stage’ analysis, the coefficients were similar to those in the main model, apart from age (1.00, 95% CI 0.99–1.01, *P* = 0.683) (see Supplementary Material).

## Discussion

### Summary

This is the first primary-care study to examine the association between pre-existing medical conditions and the stage of oesophagogastric cancer at diagnosis. We provide some evidence of differing effects of multimorbidity between the sexes. For men, being multimorbid reduces the chance of advanced-stage oesophagogastric cancer to levels seen for women regardless of their multimorbidity status, controlling for age, deprivation and cancer site. Having alternative explanations for features of possible oesophagogastric cancer is not associated with stage.

### Strengths and limitations

The study analysed 1749 primary care patients in England, ensuring ample power. Our data source was the CPRD, which has NCRAS linkage, and is large and representative, enabling generalizability of our results (20). The demographics of our sample are consistent with the epidemiology of oesophagogastric cancer, in terms of age at diagnosis, sex and deprivation profiles, and unknown stage (2,3). A further strength is the primary-care setting, where general practitioners face the difficulty of recognizing which patients need referral or investigation for undiagnosed cancer.

Our main limitations relate to missing CPRD and NCRAS data. First, when using CPRD data, we depend on how GPs choose which clinical details to record and their recording method. Our use of QOF conditions likely minimizes any underestimation of multimorbidity, as QOF conditions are well-defined and coded recording is encouraged by linkage to practice payments (23). Test results have automated transmission, with have low levels of missingness (20). However, some symptoms may have been omitted, or recorded in the ‘free-text’ section—which is inaccessible to researchers. For vague symptoms especially, up to one-third of affected participants may have symptom records ‘lost’ in free text (25). Symptom under-recording may arise because of time-pressured consultations, cognitive factors influencing clinical assessment and diagnostic reasoning (26,27). We may have underestimated the numbers with alternative explanations, reducing our power to identify associations between alternative explanations and stage.

Our study is limited by having to exclude 695 (28.4%) of the sample with no recorded stage. Incomplete staging data for oesophagogastric cancer is a known issue (2,3). Stage was plausibly missing at random, conditional on increasing age and female sex. This is consistent with patients who are elderly or who present as an emergency (more likely in women) having less comprehensive diagnostic and staging investigations (13,28,29). It is reassuring that the distributions of age and sex in our sample included in the analyses are very similar to national figures (2,3), suggesting that any bias was small. Furthermore, in sensitivity ‘missing-is-advanced’ analyses, the coefficients for multimorbidity, sex and their interaction were similar in size to those in the main model.

### Comparison with previous literature

Previous research established associations between multimorbidity and advanced-stage diagnosis of ovarian, laryngeal, breast and colorectal cancers (12,14–16). Findings related to gastric/liver cancers were inconclusive, although separate effects for gastric and liver cancers or for men and women were not investigated (19). Our findings are consistent with the surveillance hypothesis effect of multimorbidity in oesophagogastric cancer for men (7,13). Men consult primary care less frequently than women, after allowing for women’s consultations for reproductive health care; however, the difference was not observed between men and women with comparable morbidities (30).

We found no association between stage and having alternative explanations; therefore, our study does not elucidate why women are more likely than men to be diagnosed following an emergency presentation (2,3,5).

Our findings contrast with our previous study of bladder cancer (14). For that cancer, we found no association between count of pre-existing conditions and stage, but a strong association between alternative explanations for features of bladder cancer and advanced-stage diagnosis (14). This difference may reflect the symptomatology of the two cancers: haematuria is the dominant symptom and highly predictive of bladder cancer (14,31), and is recommended for urgent investigation (4). In contrast, in our study, less than one-third of oesophageal cancers presented with the high-risk symptom of dysphagia, the majority presenting with low-risk features. It may be that the increased primary-care attendances trigger investigation of low-risk symptoms, such as dyspepsia, though not necessarily with cancer as the condition being sought. There is a clear relationship between a primary-care practice’s increased gastroscopy rates and improved oesophageal cancer outcomes, supporting this interpretation (32).

### Implications for research and practice

This study is consistent with the surveillance hypothesis, at least in men. It has always been considered good primary-care practice to extend the consultation beyond the presenting complaint, by elucidating other concerns, offering health care advice and preventative care. These ‘supplementary’ aspects require time, which is not always available within a single consultation. This study cannot provide a definite mechanism for the findings, being observational, but suggests that extra patient contact may have a measureable benefit. Further planned research will explore possible mechanisms by investigating gender differences in the recognition of cancer symptoms, and assessment of cancer risk in multimorbidity.

## Supplementary Material

cmaa132_suppl_Supplementary_Materials

## Data Availability

The anonymized patient data from this study are not available due to legal privacy restrictions enforced by the CPRD. Code lists and symptom libraries are available from the authors by request.

## Declaration

Funding: MQ received a grant from the USF Health Morsani College of Medicine, Research, Innovation & Scholarly Endeavors. The dataset came from a separate Cancer Research UK-funded study on diagnostic intervals (grant reference number 21550). SP was funded by the National Institute for Health Research (NIHR) Policy Research Programme, conducted through the Policy Research Unit in Cancer Awareness, Screening and Early Diagnosis Unit. The views expressed are those of the author(s) and not necessarily those of the NIHR, The Department of Health and Social Care, Cancer Research UK, NHS or other government departments or arm’s length bodies. This research is also linked to the CanTest Collaborative, funded by Cancer Research UK (C8640/A23385), of which WH is co-Director.

Ethical approval: ethical approval for the use of anonymized electronic patient records was granted from the Clinical Practice Research Datalink’s (CPRD) Independent Scientific Advisory Committee board—protocol 16_037. Additional ethical approval for the work undertaken was given by the University of South Florida, Institutional Review Board—protocol 00034698. The study was performed in accordance with the Declaration of Helsinki.
