# Supplementary material for: Quantifying the impact of pre-existing conditions on the stage of oesophagogastric cancer at diagnosis: a primary care cohort study using electronic medical records
Source: Fam Pract. 2020 Dec 21;38(4):425–31. doi: 10.1093/fampra/cmaa132 (PMC8414906; doi:10.1093/fampra/cmaa132)
Supplement: cmaa132_suppl_Supplementary_Materials [file cmaa132_suppl_Supplementary_Materials.docx]

**Online supplementary material**

**Quantifying the impact of pre-existing conditions on the stage of oesophagogastric cancer at diagnosis: a primary care cohort study using electronic medical records**

Authors: Myra Quiroga et al.

Table S1. Features of oesophagogastric cancer in participants excluded for missing stage

| Dysphagia (87/427, 20.4% in men; 54/427, 20.1% in women) |
| --- |
| Anaemia (83/427, 19.4% in men; 65/268, 24.2% in women) |
| Upper abdominal pain (66/427, 15.5% in men, 25/268, 9.3% in women) |
| Dyspepsia/reflux (65/427, 15.2% in men, 30/268, 11.2% in women). |
| Weight loss, vomiting, nausea and haematemesis were relatively rare. |

Table S2 Sensitivity analysis (missing stage == advanced stage (n=2,444)

| Explanatory variable | Sensitivity analyses (N=2,444) | | | Main analyses (N=1,749) | | |
| --- | --- | --- | --- | --- | --- | --- |
|  | Odds ratio | 95% CI | p | Odds ratio | 95% CI | p |
| Multimorbidity, main effect | 0.62 | 0.46–0.82 | 0.001 | 0.63 | 0.47–0.85 | 0.002 |
| Sex, main effect | 0.63 | 0.42–0.93 | 0.022 | 0.59 | 0.39–0.89 | 0.011 |
| Interaction term (multimorbidity × sex) | 1.88 | 1.17–2.99 | 0.008 | 1.76 | 1.08–2.86 | 0.024 |
| Has an “alternative explanation” | 1.07 | 0.81–1.41 | 0.632 | 1.07 | 0.80–1.43 | 0.631 |
| Cancer site | 1.10 | 0.89–1.35 | 0.373 | 1.21 | 0.97–1.50 | 0.09 |
| Age at diagnosis | 1.00 | 0.99–1.01 | 0.683 | 0.99 | 0.98–1.00 | 0.004 |
| Deprivation quintile | 1.03 | 0.95–1.10 | 0.511 | 1.04 | 0.96–1.13 | 0.304 |
